# Real-time continuous glucose monitoring in the ICU

**DOI:** 10.1186/cc14453

**Published:** 2015-03-16

**Authors:** J Gios, B Manuel-y-Keenoy, P Rogiers

**Affiliations:** 1 University Hospital Antwerp, Edegem, Belgium; 2 University of Antwerp, Wilrijk, Belgium; 3 Ziekenhuisnetwerk Antwerpen Middelheim General Hospital, Antwerp, Belgium

## Introduction

Hyperglycemia occurs in 50 to 85% of patients admitted to a medical ICU (MICU) and has been associated with poor prognosis [1,2]. Whether applying intensive insulin therapy to achieve tight glycemic control in critically ill patients is beneficial remains controversial [2]. Another important observation is a link between glycemic variability and mortality [3]. We performed a pilot study hypothesizing that when implementing intensive insulin therapy, real-time continuous glucose monitoring (RT-CGM) may help to safely achieve tight glucose control, while avoiding hypoglycemia and reducing glycemic variability in MICU patients.

## Methods

This two-center randomized controlled pilot study was performed during a 3-year period. To be included, patients had to be severely ill (APACHE II score ≥20) and CGM monitoring had to be started within 48 hours after admission in the MICU. Thirty-five patients (age 66 ± 10 years; nondiabetic/diabetic patients 27/8; APACHE II score 28 ± 6) were randomly assigned to RT-CGM (*n *= 16) or to blinded CGM. In both groups a microdialysis-based glucose sensor (GlucoDay®S) was used during a 96-hour period of glucose monitoring. Insulin infusion was performed using a modified Yale protocol. Outcome measures were percentage of time in normoglycemia and in hypoglycemia, glycemic variability, and CGM accuracy.

## Results

In the RT-CGM group the percentage of time at the target glycemia (80 to 110 mg/dl) was 37 ± 12% versus 34 ± 10% in the control group (NS) and glycemia averaged 119 ± 17 mg/dl versus 122 ± 11 mg/ dl respectively (NS). Time spent in hypoglycemia (<60 mg/dl) was not statistically different between the group assigned to RT-CGM (0.6 ± 1.6% of the time) versus those with blinded CGM registration (2.4 ± 4.3% of the time). Parameters of glucose variability (standard deviation of mean glucose value, coefficient of variation, mean amplitude of glucose excursions) did not differ between the groups. The GlucoDay®S values and arterial glycemia correlated well with 98.6% of data falling in regions A and B of error grid analysis.

## Conclusion

In our study, the use of RT-CGM neither improved glucose control and variability, nor did it reduce hypoglycemic events. On the other hand we can state that our insulin infusion protocol already led to overall tight glucose control without a significant hypoglycemia risk, leaving little space for improvement.
